# Semantic validation of the use of SNOMED CT in HL7 clinical documents

**DOI:** 10.1186/2041-1480-2-2

**Published:** 2011-07-15

**Authors:** Stijn Heymans, Matthew McKennirey, Joshua Phillips

**Affiliations:** 1 SemanticBits, LLC, 13921 Park Center Road Suite 420, Herndon, VA 20171, USA

## Abstract

**Background:**

The HL7 Clinical Document Architecture (CDA) constrains the HL7 Reference Information model (RIM) to specify the format of HL7-compliant clinical documents, dubbed *CDA documents*. The use of clinical terminologies such as SNOMED CT^® ^further improves interoperability as they provide a shared understanding of concepts used in clinical documents. However, despite the use of the RIM and of shared terminologies such as SNOMED CT^®^, gaps remain as to how to use both the RIM and SNOMED CT^® ^in HL7 clinical documents. The HL7 implementation guide on *Using SNOMED CT in HL7 Version 3 *is an effort to close this gap. It is, however, a human-readable document that is not suited for automatic processing. As such, health care professionals designing clinical documents need to ensure validity of documents manually.

**Results:**

We represent the CDA using the Ontology Web Language OWL and further use the OWL version of SNOMED CT^® ^to enable the translation of CDA documents to so-called OWL *ontologies*. We formalize a subset of the constraints in the implementation guide on *Using SNOMED CT in HL7 Version 3 *as OWL *Integrity Constraints *and show that we can automatically validate CDA documents using OWL reasoners such as Pellet. Finally, we evaluate our approach via a prototype implementation that plugs in the Open Health Workbench.

**Conclusions:**

We present a methodology to automatically check the validity of CDA documents which make reference to SNOMED CT^® ^terminology. The methodology relies on semantic technologies such as OWL. As such it removes the burden from IT health care professionals of having to manually implement such guidelines in systems that use HL7 Version 3 documents.

## Background

### Introduction

*Health Level Seven International (HL7) *[1] is a non-profit organization that develops standards to increase the interoperability of health care information technology. One such standard is the *Reference Information Model (RIM) *[2] that functions as the common information model for all further specified information models and messages developed under the auspices of HL7. For example, the HL7 standard for writing clinical documents is provided by the *Clinical Document Architecture (CDA) *[3] and is a constraining of the RIM. It specifies how HL7 clinical documents should be structured while using classes and attributes defined in the RIM.

The advantage of using such a layered architecture of models for different purposes is its provision of syntactic (and to a certain extent semantic) interoperability throughout the health care domain. Clinical documents for exchange between health care professionals that satisfy this CDA (CDA documents), are syntactically understandable by both ends of the exchange. The CDA caters for some semantic interoperability. Indeed, it prescribes what meta-vocabulary to use in the form of directions such as to use the class *SubstanceAdministration *for administering a substance. However, an extra factor is needed to understand also the particular data being sent in such clinical documents.

Clinical terminologies such as SNOMED CT^® ^provide the means for standardizing such data. If a health care professional references a CDA *SubstanceAdministration *of a SNOMED CT^® ^concept with ID *433181003*, the receiving professional knows that *Amoxicillin 775 mg extended release tablet *is targeted for *SubstanceAdministration*. Note that such clarity (or semantic interoperability) is important for humans, but becomes an even more pressing issue if the clinical documents are to form the basis of automated decision making: is the substance administration of Amoxicillin appropriate given the exhibited symptoms?

Even though a combination of the HL7 RIM and the use of standardized clinical terminologies goes a long way toward semantic interoperability, exactly their use together can lead to interoperability problems. Indeed, there is a certain overlap between the semantics of the RIM and the semantics of SNOMED CT^® ^codes. A SNOMED CT^® ^code may express a meaning that the RIM expresses using a combination of classes [4].

To clarify the use of SNOMED CT^® ^in the context of HL7 documents, guidelines are established in [4]. The guidelines indicate, among others, what the code of a certain CDA *Observation *should be if a SNOMED CT^® ^concept is used (it should be a subtype of the SNOMED CT^® ^concept *Observable entity*). These guidelines are written in natural language and at present no automated means is available to check whether a clinical document satisfies them to ensure that both sender and receiver can interpret the result the same way.

The goal of this work is to verify how to automatically validate CDA documents for satisfaction of the guidelines on the use of SNOMED CT^® ^in HL7 documents.

We provide a possible solution for this problem by using the Web Ontology Language OWL [5]. OWL is a W3C standard for representing knowledge on the Web, and serves in general as a language for expressing *ontologies*, where ontologies were conceived as formalized representations of knowledge that provide a "shared understanding" [6] of certain domains. Moreover, one can reason over representations in OWL: one can determine which concepts are equivalent, which are subsumed, and whether the knowledge base is inconsistent. As such it is an excellent candidate for a lingua franca in the health care domain, and able to inject the necessary automatization for semantic interoperability. We show that by writing the available knowledge in the current scenario in OWL and by using OWL reasoners such as Pellet, we can exactly tackle the question *does my CDA document satisfy the guidelines on the use of SNOMED CT^®^?*

This article reports on how to write OWL representations of the different knowledge involved in the above problem when dealing with CDA documents. We define an ontology for the CDA, use the OWL version of SNOMED CT^®^, write a specific subset of the guidelines (the SNOMED CT^® ^vocabulary domain constraints [[4], Section 5]) as OWL, and show how to transform CDA documents to OWL ontologies. We then indicate how to use automated reasoning via ontology reasoners to validate CDA documents for conformance to the usage of SNOMED CT^®^. We further report on a prototype implementation that extends the Open Health Workbench [7] with a plug-in for such semantic validation. Finally, we discuss some common errors that we found in CDA documents with respect to to the SNOMED CT^® ^guidelines and we identify areas of future work.

### Methods

#### Reasoning with OWL Integrity Constraints

One of the corner stone ideas of the envisioned Semantic Web [8] is to move from human-readable content on the Web to machine-processable content that can be automatically reasoned about. Layered on top of basic Web technologies such as XML, RDF [9], RDF(S) [10], the *OWL Web Ontology Language *[5], and its expressive successor *OWL 2 *[11] provide for an expressive logic-based representation of knowledge on the Web.

Expressive fragments of OWL, the so-called *OWL DL *fragments, correspond directly to a particular *Description Logic *[12]. Traditionally, Description Logics form a set of logical languages that balance complexity and expressiveness: they are usually decidable fragments of undecidable first-order logic with a syntax that has as basic building blocks *concepts *and *relationships or roles*. In contrast with first-order logic, they allow for decidable reasoning and can handle tasks such as verifying whether one concept is subsumed by another concept or whether concepts are satisfiable.

As such, Description Logics are recognized also in the health care domain as a useful framework to build clinical terminologies. SNOMED CT^® ^(see below) is defined as a particular Description Logic theory, allowing users to, for example, identify redundancy by querying for clinical concepts that are equivalent [13].

We use in this article the Manchester OWL syntax [14] as it is easy for humans to read and close to the underlying Description Logics syntax. we refrain from defining the syntax in detail but will explain its intuition whenever we use it. Take the following example:(1)

The basic building blocks are the concepts *PCNAllergy *and *Penicillin *which are used to classify individuals and their type. The basic relationship or role used is *causativeAgent *which relates individuals to other individuals, namely its causative agents. The concept expression (*causativeAgent ***some ***Penicillin*) is called an *exists restriction *as it captures all individuals that have *some *causative agent that is a penicillin. *Axiom *(1) then imposes that every individual that is of type *PCNAllergy *also needs to have a *causativeAgent *relation with some individual that is of type *Penicillin*.

We can make this axiom stronger by writing:(2)

Instead of a **SubClassOf **axiom this is an **EquivalentTo **axiom and can be seen as 2 **SubClassOf **axioms: axiom (1) and(3)

It indicates that individuals of type *PCNAllergy *are exactly those allergies that have a causative agent that is penicillin.

The semantics of such sets of OWL axioms, called *ontologies*, is given by first-order interpretations and thus obeys the *open world assumption*. This open world assumption has important consequences on the type of reasoning possible with OWL. Assume we have a particular individual *i *that is of type *PCNAllergy*. Denoted in traditional first-order logic notation, we have *PCNAllergy *(*i*). With the **SubClassOf **axiom (1), we deduce that there exists some individual *j *such that *causativeAgent *(*i, j*) and *Penicillin*(*j*) holds. Given this open world semantics, if such a *j *does not exist explicitly, it is assumed to exist implicitly to satisfy the axiom. In the case of reasoning with domain knowledge such as clinical terminologies such standard logical entailment allows to answer questions such as "When my patient has an allergy, should this be associated with a causative agent?" The usual semantics says "yes, there is *some *causative agent."

In the context of pure conceptual reasoning such an open world assumption is suitable and desirable (one does not want to include a representative data set to test equivalence of two concepts). However, when data is present that needs to conform to an ontology, one would like the axioms to behave more like *integrity constraints (ICs)*, i.e., one would like to treat OWL as a schema language for the instance data. In the above example, a data set containing only *PCNAllergy*(*i*) should lead to a violation of the *PCNAllergy *axiom. A data set containing on the other hand *PCNAllergy*(*i*), *causativeAgent*(*i, j*) and *Penicillin*(*j*) is not violating the axiom. Indeed, whereas logical entailment is useful for reasoning over the domain knowledge, when dealing with clinical documents, one does not just want to know that there is a sole, possibly unknown, causative agent. One wants to verify that the clinical document is explicitly mentioning this causative agent.

In [15] such an integrity constraint semantics for OWL axioms is defined, enabling OWL axioms to be used as constraints that must be satisfied by particular instance data, thus effectively implementing a closed world assumption. An implication for our treatment of CDA documents is that, whereas the usual semantics of OWL is interested in what can be inferred, the integrity constraint semantics cares about what is or is not in the document. A prototype integrity constraint validator, Pellet-ICV [16], is implemented based on the Pellet OWL reasoner [17].

SNOMED CT

The *Systematized Nomenclature of Medicine *- *Clinical Terms *(SNOMED CT^®^) [18] is a reference terminology for clinical data. Such a clinical reference terminology is described by [13] as

"[. . .] a set of concepts and relationships that provides a common reference point for comparison and aggregation of data about the entire health care process, recorded by multiple different individuals, systems, or institutions. The main purpose of a reference terminology for clinical data is the retrieval and analysis of data relating to the causes of disease, the treatment of patients, and the outcomes of the overall health care process."

The work described in this paper uses the SNOMED CT^® ^core International Release of January 2010 [18], which consists of 291144 concepts. For example, the concept *Amoxicillin 775 mg extended release tablet (product) *has a concept identifier *433181003 *and is indicated by SNOMED CT^® ^to be a *350162003 *|*Oral form Amoxicillin (product) *where we use the *conceptId *|*Fully Specified Name *format for SNOMED CT^® ^concepts. Moreover, it has as an *active ingredient *the concept *372687004 *|*Amoxicillin (substance)*. Note that also relationships between concepts have an identifier: *has active ingredient *has the identifier *127489000*. For readability, we will refrain from using SNOMED CT^® ^concept identifiers or fully specified names. Instead, we will use short human-readable names throughout the paper where appropriate.

Such stated relationships of SNOMED CT^®^, i.e., the clinical statements that are directly defined by authors or editors, in contrast with inferred relationships, can be translated to the ontology language OWL to enable specific inferencing such as determining the equivalence of concepts or hierarchical relationships between concepts. The *SNOMED CT Stated Relationships Guide *[19] describes a script that provides exactly this translation.

In Table 1, we extend the above example that shows how SNOMED CT^® ^indicates that *Amoxicillin *is a particular *Penicillin *and that an *Allergy to penicillin *has *Penicillin *as a causative agent.

**Table 1:** SNOMED CT^® ^Fragment Amoxicillin

(1)	*Amoxicillin Tablet ***Has active ingredient ***Amoxicillin*
(2)	*Amoxicillin ***Is a ***Aminopenicillin*
(3)	*Aminopenicillin ***Is a ***Penicillin*
(4)	*PCNAllergy ***Causative agent ***Penicillin*

The equivalent OWL representation of Table 1 is listed in Table 2. The *IsA *relations are directly translated using the **SubClassOf **construct of OWL, while other relationships are defined using OWL exists restrictions: the expression (HasActiveIngredient **some ***Amoxicillin*) collects all individuals that have some active ingredient that is a *Amoxicillin*. Axiom (1) in Table 2 then indicates that every individual that is a *AmoxicillinTablet *is also an individual that has some active ingredient that is a *Amoxicillin*.

**Table 2:** OWL Representation SNOMED CT^® ^Fragment Amoxicillin

(1)	*AmoxicillinTablet ***SubClassOf **(HasActiveIngredient**some ***Amoxicillin*)
(2)	*Amoxicillin ***SubClassOf ***Aminopenicillin*
(3)	*Aminopenicillin ***SubClassOf ***Penicillin*
(4)	*PCNAllergy ***SubClassOf **(RoleGroup**some **(causativeAgent**some ***Penicillin*))

The only non-trivial axiom is axiom (4) that uses the special non-SNOMED CT^® ^role *RoleGroup*. This role is used to group OWL exists restrictions together (see [20] for more details on role groups), and essentially has in this context the same effect as the earlier axiom describing the causative agent of a Penicillin allergy.

#### Clinical Statements in the CDA

The HL7 Clinical Document Architecture, Release 2 (CDA) [3] is a standard that prescribes the structure and semantics of document markup for clinical documents such as discharge reports and progress reports. The CDA is a derivation of the HL7 Reference Information Model (RIM) [2] which enables it to refer to external code systems such as SNOMED CT^®^. CDA documents are particular instances conforming to this CDA and can contain text, images, and referrals to particular codes in HL7-endorsed code systems such as SNOMED CT^®^. In this work, we focus on a particular fragment of the CDA, the *Clinical Statement *pattern, which specifies the structure and semantics of typically used RIM Acts in CDA documents such as *Observation*, *SubstanceAdministration*, *Supply*, *Procedure*, *Encounter*, *Organizer*, and *Act*.

Consider the following fragment of a CDA document from [4]:

1 <observation classCode="OBS" moodCode="EVN">

2    <code code="ASSERTION" codeSystem="2.16.840.1.113883.5.4"/>

3    <text>Allergy to PCN manifesting as hives </text>

4    <value xsi:type="CD" code="106190000|Allergy|:246075003|Causative agent|=373270004| Penicillin - class of antibiotic - (substance)" codeSystem="2.16.840.1.113883.6.96"/>

5    <actRelationship typeCode="MFST "inversionInd="true" contextConductionInd="true">

6       <observation classCode="OBS" moodCode="EVN">

7          <code code="ASSERTION" codeSystem="2.16.840.1.113883.5.4"/>

8          <value xsi:type="CD" code="247472004| Hives|" codeSystem="2.16.840.1.113883.6.96">

9             <displayName value="Hives"/>

10          </value>

11       </observation>

12    </actRelationship>

13 </observation>

Without going into details (see [3] for more on CDA documents) the fragment consists of an *Observation *of an allergy to penicillin manifesting as hives. It has moodCode="EVN" indicating that the observation has occurred and has a particular code indicating that the observation is an ASSERTION, where ASSERTION is in turn a code from the code system 2.16.840.1.113883.5.4, the HL7 *ActCode *code system.

The value of the observation is taken from the code system SNOMED CT^®^, identified by 2.16.840.1.113883.6.96, and is of data type CD, an HL7 defined data type that allows for the combination of codes from a code system to define specific concepts. Indeed, the value uses different codes from SNOMED CT^® ^to describe the so-called *post-coordinated *expression 106190000| Allergy| :246075003|Causative agent|=373270004| Penicillin - class of antibiotic - (substance), an allergy that has penicillin as a causative agent.

The actRelationship on line 5 with typeCode="MFST" relates this observation subsequently with the observation on line 6, its manifestation. This observation is similarly defined as an ASSERTION with a value code from SNOMED CT^® ^that represents hives.

#### Guidelines on Using SNOMED CT^® ^in HL7 CDA Documents

The use of external code systems such as SNOMED CT^® ^in HL7 specifications is one aspect of achieving the HL7 goal of semantic interoperability: not only can different parties understand clinical information syntactically, they also agree on the meaning of this clinical information. By committing to widespread ontologies such as SNOMED CT^®^, each party knows what is meant when using particular SNOMED CT^® ^terminology.

However, when representing clinical information conforming to a reference model like the HL7 RIM, just using SNOMED CT^® ^codes is not sufficient to avoid semantic mismatches. For example, the HL7 CDA *Observation *class has 2 attributes, *Observation.code *and *Observation.value*: how to use these attributes to represent clinical findings, i.e., results of a clinical observation, such as *has a fracture of her left femur*? One could use the *code *attribute to encode this statement via a SNOMED CT^® ^expression, but it would be unclear how the *value *attribute should be assigned, and vice versa.

The set of guidelines presented in *Using SNOMED CT in HL7 Version 3; Implementation Guide, Release 1.5 *[4] addresses issues like the above: how to use SNOMED CT^® ^in the context of HL7 clinical statement patterns? Clinical statement patterns [21] are constraints on the HL7 RIM and occur in the CDA. In the example above, the *code *for the observation can be an *ASSERTION *and the *value *can be a SNOMED CT^® ^expression for *has a fracture of her left femur *which is a subexpression of the clinical finding SNOMED CT^® ^concept.

We will focus in this paper on the guidelines around the *SNOMED CT^® ^vocabulary domain constraints *[[4], Section 5] and this in context of the clinical statement pattern in the CDA. Note that the approach we present here can be applied to clinical statement patterns in general.

Consider a constraint from [[4], Section 5] in Table 3. It indicates that for *Observation *classes with class code *OBS*, and if a SNOMED CT^® ^expression is used for the *Observation.value *(this is not explicitly present in Table 3. It is, however, a prerequisite for the constraints to be applicable) and the *Observation.code *is *ASSERTION*, then the *Observation.value *should be a subclass of the SNOMED CT^® ^concept *Clinical Finding *or of *Finding with Explicit Context *or of *Event*. The '<<' in front of a SNOMED CT^® ^expression represents all concepts that are subclasses of that expression or the expression itself.

**Table 3:** Constraint on *Observation.value *when *Observation.code *is *ASSERTION*

**Class Name**	Observation
**Class Code**	OBS
**Attribute Name**	Observation.value
**Narrative description**	An act that is intended to result in new information about a subject. The main difference between observations and other acts is that it has a value attribute that is used to state the result of the assessment action described in Act.code.
**Simple representation**	((<<404684003| clinical finding|)OR(<<413350009| finding with explicit context|)OR (<<272379006| event|))
**Notes**	Where Observation.code = ASSERTION. An alternative approach (now deprecated) is for the same value set to be communicated in Observation.code where the attribute Observation.value is not present in the Observation class instance. As indicated in section 2.2.2.2 subheading 7, the situation may arise in which Observation.value is a SNOMED CT expression from the set specified in the 'simple representation' field of this table and Act.code is represented by a code other than ”ASSERTION”. For such an approach can only be safely used if interpretation of the Act.code together with the Observation.value does not yield a meaning that is substantially different from the meaning implied if the Act.code was ”ASSERTION”. Without exhaustive scrutiny of SNOMED CT's content it is not possible to identify that set of codes that can safely be used in this way in Act.code.

## Used Method

We show how to verify a given CDA document with respect to the guidelines for the use of SNOMED CT^®^, in other words, we test if the CDA document satisfies these guidelines?

**Figure 1:**
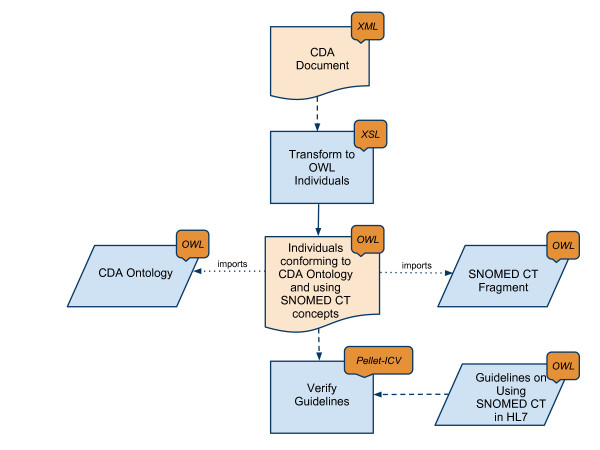
**Used Method Verifying Guidelines SNOMED CT^®^**. The figure describes a top level view of the used method to validate CDA documents using semantic technologies.

As illustrated in Figure 1 our method consists of transforming, by means of an XSL transformation, a particular CDA document in XML syntax to a set of OWL individuals using a CDA ontology and the SNOMED CT^® ^OWL representation. We further write the constraints on the use of SNOMED CT^® ^from [[4], Section 5] as OWL axioms and then test the consistency of those OWL axioms with respect to the set of OWL individuals that represent the original CDA document.

### Constructing the CDA Ontology

In order to write CDA documents as particular OWL individuals, specifically for testing CDA documents for conformance to the SNOMED CT^® ^guidelines, we define an ontology representing the relevant parts of the clinical statement pattern of the CDA. Based on the RIM in Figure three of [3], we model, for example, the CDA *Observation *class as in Table 4.

**Table 4:** OWL Representation of the CDA *Observation *Class

(1)	*Observation ***SubClassOf **classCode **some ***OBS*
(2)	*Observation ***SubClassOf **classCode **only ***OBS*
(3)	*Observation ***SubClassOf **moodCode **some ***x*_ActMoodDocumentObservation
(4)	*Observation ***SubClassOf **moodCode **only ***x*_ActMoodDocumentObservation
(5)	*Observation ***SubClassOf **hasValue **only ***HL7SupportedCodeSystems ***or ***HL7ValueSets*
(6)	*Observation ***SubClassOf **hasCode **only ***HL7SupportedCodeSystems ***or ***HL7ValueSets*

We note the use of the concepts *HL7SupportedCodeSystems *and *HL7ValueSets *in axioms (5) and (6). They indicate that CDA *Observations *have values and codes that come either from an HL7 supported code system or from an HL7 value set. An example of such an HL7 supported code system is SNOMED CT^® ^or the HL7 *ActClass *code system. Indeed, besides the classes appearing in the CDA clinical statement pattern, we also add to this ontology the particular HL7 supported code systems with their hierarchically organized codes and the HL7 value sets that are defined using those codes. For example, Table 5 describes the concept code *OBS *as a subclass of the concept code *ACT*, a subclass of the code system *ActClass *that is in turn a subclass of the placeholder concept *HL7SupportedCodeSystems*.

**Table 5:** HL7 Supported Code Systems Fragment

(1)	*OBS ***SubClassOf ***ACT*
(2)	*ACT ***SubClassOf ***ActClass*
(3)	*ActClass ***SubClassOf ***HL7SupportedCodeSystems*

The value sets are defined as hierarchies as well and subclass the placeholder concept *HL7ValueSets*. They are defined using concepts that subclass from *HL7SupportedCodeSystems *as can be seen in a fragment in Table 6. There, *x_ActMoodDocumentObservation *is defined as equivalent to any of the concept codes in HL7 supported code systems on the right-hand side of axiom (1). It additionally is indicated to be a subclass of the placeholder for value sets using the coding system *ActMood *which in turn is a subclass of the placeholder concept *HL7ValueSets*.

**Table 6:** HL7 Value Sets Fragment

(1)	*x*_ActMoodDocumentObservation **EquivalentTo ***DEF ***or ***EVN ***or ***GOL ***or ***INT ***or ***PRMS ***or ***PRP ***or ***RQO*
(2)	*x_ActMoodDocumentObservation ***SubClassOf ***VSUsingActMood*
(3)	*VSUsingActMood ***SubClassOf ***HL7ValueSets*

Reconsidering Table 4, one sees that axiom (1,2) and (3,4) indicate that CDA *Observations *should have a class code that is of type *OBS *and a mood code that is of type *x_ActMoodDocumentObservation *respectively. Axiom (1) in Table 6 indicates that a mood code that is of type *EVN *would thus be allowed as the mood code for CDA *Observations*.

The used CDA ontology can be found as Additional file 1 or at http://stijnheymans.net/ontologies/CDA_ClinicalStatement_for_TermInfo.txt.

### Transforming the CDA Document to OWL Individuals

Reconsider the earlier XML fragment of the CDA document. Note the presence of 2 CDA *Observations*, each with a class code OBS, a mood code EVN, a code, and a value. For each of those 2 observations, we introduce 2 OWL individuals and we use the previously introduced CDA ontology to define their class and mood code, as well as the code and value for those observations. Table 7 lists the OWL individuals for the first observation.

**Table 7:** OWL Individuals for CDA Observations

Individual:	Obs_pcn_allergy
Types:	*Observation*
Facts:	classCode class_code_obs1
	moodCode mood_code_obs1
	hasCode code_obs1
	hasValue value_obs1

Note the definition of the individual obs_pcn_allergy that is of type *Observation *(a concept from the CDA ontology), and is related via the role classCode (also defined in the CDA ontology) to the individual class_code_obs1 that represents the class code. The individuals class_code_obs1, mood_code_obs1, code_obs1, and value_obs1 are in turn defined appropriately using the CDA ontology as in Table 8.

**Table 8:** OWL Individuals

Individual:	class_code_obs1
Types:	*OBS*
Individual:	mood_code_obs1
Types:	*EVN*

Individual:	Code_obs1
Types:	*ASSERTION*

Individual:	value_obs1
Types:	*Allergy ***and** (RoleGroup**some **(causativeAgent**some ***Penicillin*))

Note that the concept *ASSERTION *is a concept code in the HL7 *ActCode *code system where the latter is an HL7 supported code system and thus present in our defined CDA ontology. Further note how the post-coordinated expression 106190000| Allergy|:246075003|Causative agent|=373270004| Penicillin - class of antibiotic - (substance) was rewritten as the OWL expression using the RoleGroup role and concepts from the SNOMED CT^® ^OWL ontology: *Allergy ***and **(RoleGroup**some **(causativeAgent**some ***Penicillin*)).

The use of OWL expressions that are constructed using SNOMED CT^® ^concepts implies that to write a CDA document as a set of individuals, we import not only the CDA ontology but also the SNOMED CT^® ^OWL ontology. We further correctly connect SNOMED CT^® ^with the CDA ontology by making the top SNOMED CT^® ^concept *138875005 *|*SNOMED CT Concept (SNOMED RT+CTV3) *a subclass of the placeholder concept *SNOMEDClinicalTerms *(in turn a subclass of the *HL7SupportedCodeSystems *concept) in the CDA Ontology.

The reader can find the used fragment of SNOMED CT^® ^in Additional file 2 or at http://stijnheymans.net/ontologies/SNOMED_CT_for_TermInfo.txt.

Note that we do not model the actRelationship as the SNOMED CT^® ^guidelines are scoped to isolated CDA classes. As such the OWL translation of a CDA document is not lossless: we lose the structure of the CDA documents, which, on the other hand, causes the OWL individuals ontology to have a simple at structure.

### The Guidelines on Using SNOMED CT^® ^in HL7 CDA Documents as OWL Integrity Constraints

We need one more component to be able to verify CDA documents for conformance with the guidelines on using SNOMED CT^®^: the guidelines written as OWL Integrity Constraints.

Take, for example, the constraint expressed in Table 3, which we can phrase as

If an *Act *has a class code *OBS *(i.e., it is an *Observation*), and the *Observation*'s code is *ASSERTION*, and SNOMED CT^® ^is used to provide a value for the *Observation*, then this possibly post-coordinated expression has to be either a *404684003 *|*clinical finding *or a *413350009 *|*finding with explicit context *or a *272379006 *|*event*.

We write this constraint as the following OWL integrity constraint:(4)

The first component of this integrity constraint, (valueOf**some **(*Observation ***and **(hasCode**some ***ASSERTION *))), picks up the individuals that are the value of some CDA *Observation *(valueOf is the inverse of hasValue in the CDA ontology) where the CDA *Observation *has a code that is an *ASSERTION*. These individuals additionally have to be related via the codeSystem role to the individual 2.16.840.1.11388.3.6.96 which represents the SNOMED CT^® ^code system. The **SubClassOf **then indicates that such individuals need to be either of type *ClinicalFinding*, *FindingWithContext*, or *Event*.

The other constraints in [[4], Section 5] can be written similarly as OWL integrity constraints. The reader can find the integrity constraints in Additional file 3 or at http://stijnheymans.net/ontologies/TermInfo_ICs.txt.

### Verification of the Guidelines

Reconsider the example CDA document in Tables 7 and 8. Is this document satisfied by the constraints on the use of SNOMED CT^®^?

Take, for example, the integrity constraint (4). The individual value_obs1 is the valueOf obs_pcn allergy which is of type *Observation*. Indeed, valueOf is the inverse role of hasValue and obs_pcn_allergy hasValue value_obs1. Moreover, the individual obs_pcn_allergy has a code code_obs1 that is of type *ASSERTION*. Finally, since value_obs1 is defined using concepts from the SNOMED CT^® ^ontology it inherits the codeSystem value 2.16.840.1.11388.3.6.96 from the placeholder concept *SNOMEDClinicalTerms *for SNOMED CT^® ^in the CDA ontology. Note that the ontology with OWL individuals corresponding to the CDA document imports both the CDA ontology and the SNOMED CT^® ^ontology and it appropriately makes the top SNOMED CT^® ^concept a subclass of *SNOMEDClinicalTerms *in the CDA ontology.

The individual value_obs1 thus satisfies the left-hand side of integrity constraint (4) -- left/right is with respect to the **SubClassOf **symbol. In order for the integrity constraint to be satisfied the individual value_obs1 is required to also satisfy the right-hand side, i.e., it has to be of type *ClinicalFinding ***or ***FindingWithContext ***or ***Event*. Recall from Table 8 that value_obs1 is of type *Allergy ***and **(RoleGroup**some **(causativeAgent**some ***Penicillin*)).

As the OWL individuals corresponding to the CDA document import the SNOMED CT^® ^OWL ontology, we can deduce with standard OWL reasoning that *Allergy ***and **(RoleGroup**some **(causativeAgent**some ***Penicillin*)) is a subclass of the concept *ClinicalFinding*, and thus, that value_obs1 is of type ClinicalFinding, and finally, also of *ClinicalFinding ***or ***FindingWithContext ***or ***Event*. This satisfies the integrity constraint corresponding to the SNOMED CT^® ^guideline.

Note that we intertwine standard OWL reasoning with OWL Integrity Constraint reasoning: we use standard reasoning to infer concepts in SNOMED CT^® ^and use the Integrity Constraint semantics to test the constraints. To illustrate this, assume we replace the integrity constraint (4) with the integrity constraint (5) where we leave out the clinical finding concept on the right-hand side:(5)

Again we have that value_obs1 satisfies the left-hand side of the integrity constraint. As before, and using standard OWL semantics, we can infer that value obs1 is of type *ClinicalFinding*. However, to satisfy the integrity constraint, we would need that it is of type *FindingWithContext ***or ***Event*. If we would use the standard OWL semantics we would *infer *that value_obs1 is indeed of that type in order to satisfy the integrity constraint. Using the integrity constraint semantics, the constraint is violated as we cannot infer via SNOMED CT^®^ (which we treat with standard OWL semantics) that it is of the desired type.

## Implementation

We implemented the used method to demonstrate the validation of CDA documents in terms of guidelines on the use of SNOMED CT^®^. Our implementation is based on the *Open Health Workbench *[7], an Eclipse-based tool for editing HL7 clinical documents. We added our technology to the Open Health Workbench in the form of an Eclipse plug-in. Consequently, we can open HL7 CDA documents and via a newly added validation option, we can automatically check SNOMED CT^® ^usage guidelines for this document. The output of such a violation indicates which of the HL7 guidelines was violated and assists the user in fixing the CDA document such that it does conform to the guidelines.

Under the hood, as explained above, we work with OWL as the lingua franca for our validation. As SNOMED CT^® ^provides an OWL version and we designed OWL integrity constraints for the guidelines on using SNOMED CT^® ^in CDA documents, the only remaining component for automatic transformation to OWL is the CDA document. We accomplish the automatic transformation of CDA documents by using an XSL transformation that takes the XML format supported by the Open Health Workbench and translates this on the fly to OWL individuals conforming to the CDA ontology. The actual validation incorporating the different OWL ontologies is then done using Pellet-ICV [16].

A demo demonstrating the technology is available [22].

## Results and Discussion

We tested our method on an archive of existing CDA document examples provided in [23]. A typical violation of the guidelines of using SNOMED CT^® ^in CDA documents can be seen in the below fragment from *HITSP C32: HITSP Summary Documents Using HL7 CCD *at [24]:

1 <observation classCode="OBS" moodCode="EVN">

2       <templateId root="2.16.840.1.113883.10.20.1.28" assigningAuthorityName="HL7 SDTC CCD"/>

3       <templateId root ="1.3.6.1.4.1.19376.1.5.3.1.4.5" assigningAuthorityName="IHE PCC"/>

4    <id root="0 fdf994f-2839-482d-bb92-f9b9d1a1786f"/>

5    <code code="64572001" displayName="Condition" codeSystemName="SNOMED CT"

6       codeSystem="2.16.840.1.113883.6.96"/>

7    <text >

8          <reference value="cond001"/>

9    </text >

10    <statusCode code="completed"/>

11    < effectiveTime >

12       <low value="1999"/>

13       <high value="1999"/>

14    </effectiveTime >

15    <value xsi:type="CD" code="37796009" displayName="Migraine" codeSystemName="SNOMED CT"

16       codeSystem="2.16.840.1.113883.6.96"/>

17 </observation>

Indeed, we have in [[4], Section 5.3.1.1] a vocabulary domain constraint saying that an *Ob-servation.value *should be ((<<281296001|result comments|) OR (<<260245000|findings values|)) if SNOMED CT^® ^is used to encode the value. This constraint is violated as *Migraine *is neither a subtype of the *result comments *concept nor of the *findings values *concept. Actually, the *Migraine *is a subtype of the used *Observation.code *Condition.

Additionally, this fragment also violates the constraint saying that an *Observation.code *should be ((<<386053000|evaluation procedure|) OR (<<363787002|observable entity|)), as *Condition *is neither an observable entity nor an evaluation procedure.

A possible way of resolving this issue is to use *ASSERTION *for the *Observation.code*. The *Observation.value *can then be left as is.

As a proof-of-concept, we further analyzed 26 CDA documents that are available from [23]. Note that only 14 of those 26 documents use SNOMED CT^® ^concepts in their definition, such that they are the only relevant ones for analysis. We grouped the 8 Integrity Constraints that validate the usage of SNOMED CT^® ^in the documents in 5 classes pertaining to Observations, Entities, Procedures, Substance Administrations, and Organizers. The group of Observation constraints contains 3 axioms while the other groups contain 1 violation constraint each. Table 9 shows the 5 groups in the left-hand column. The second column lists how many statements in the 14 CDA documents refer to either Observations, Entities, Procedures, Substance Administrations, Supplies, and Organizers by means of SNOMED CT^® ^concepts. The third column indicates how many of those statements were violated by the particular group of Integrity Constraints. The final column shows the percentage of violations.

**Table 9:** Initial Evaluation Results

	SNOMED CT^® ^Usages in CDA	Violated Statements	%
Observation	239	175	73%
Entity	6	0	0%
Procedure	7	0	0%
Substance Administration	2	2	100%
Organizer	4	2	50%

We note that even though the sample is not significant, 73% of the statements that use Observations using SNOMED CT^® ^are violated. The CDA documents do not make extensive use of other definitions than Observations, so other results are not indicative. Note that the example is typical for the violated constraints in this 73% group around Observations.

A remaining challenge in our approach is the usage of the full SNOMED CT^® ^ontology. Due to its sheer size this is at the moment practically impossible. In our examples as well as the prototype, we use a tailored SNOMED CT^® ^ontology that incorporates the relevant hierarchies based on the CDA document we want to validate. The tailored version defines 358 concepts and is thus significantly smaller than the full SNOMED CT^® ^which contains close to 300,000 concepts. It is part of future research to extract the relevant SNOMED CT^® ^fragment for which we will consider modular extraction techniques [25].

The current prototype supports transformation of CDA documents to OWL individuals where the CDA document contains only *simple *SNOMED CT^® ^concepts: post-coordinated expressions are not translated to their proper OWL equivalents yet. Additionally, the prototype currently uses Pellet-ICV to validate OWL Integrity Constraints. As the latter is closed source software, it is not ideal as a final solution. We are working on both issues: the translation of SNOMED CT^® ^expressions confirming to the compositional grammar to proper OWL and the usage of alternatives for Pellet ICV such as DL-programs [26] as described in [16].

Besides the SNOMED CT^® ^vocabulary domain constraints in [[4], Section 5], the implementation guide [4] provides also guidelines on the overlaps between the RIM and SNOMED CT^® ^semantics [[4], Section 2]. These overlaps are in general much harder, if possible at all, to model in OWL. Guidelines such as

"Act class clones SHALL include the priorityCode attribute if there is a requirement for expressing the urgency of a request, tracking and auditing services based on requested prioritization or any other aspects of workflow management related to priority."

are largely dependent on human intervention. Only a human can decide whether the goal is to express the urgency of a request. We intend to model as much as possible of [[4], Section 2] as integrity constraints, similarly to our treatment of all constraints in [[4], Section 5], and thus identifying a subset of the constraints that are expressible in OWL. This would provide a sense of focus for CDA document developers: which guidelines can be handled automatically, which ones need special manual care?

## Conclusions

We showed a strategy enabling automatic validation of the implementation guidelines/rules on using SNOMED CT^® ^in HL7 documents. Using the available SNOMED CT^® ^OWL representation and a Clinical Statement OWL representation, one can use OWL Integrity Constraints to automatically validate CDA documents regarding their conformance with the vocabulary domain constraints in [[4], Section 5]. As such it removes the burden from IT professionals of having to manually implement such guidelines in systems that use HL7 Version 3 documents.

## Competing interests

The authors declare that they have no competing interests.

## Authors' contributions

SH and JP devised the described used method. SH prepared a draft of the article and JP did major revisions. MM implemented the plug-in for the Open Health Workbench, scripted and built the demo, and contributed to revisions of the paper. All authors read and approved the final manuscript.

## Supplementary Material

Additional file 1 **CDA Clinical Statement Ontology for TermInfo Guidelines**. Click here for file

Additional file 2 **SNOMED CT^® ^Fragment Used in Prototype**. Click here for file

Additional file 3 **TermInfo Constraints**. Click here for file
